# Preclinical application of a dual-tracer SPECT approach with [^111^In]In-PSMA-I&T and [^99m^Tc]Tc-DPD for improved diagnosis of prostate cancer

**DOI:** 10.1186/s41181-026-00484-6

**Published:** 2026-08-14

**Authors:** Veronika Felber, Johanna Zörgiebel, Theresa Balber, Thomas L. Mindt

**Affiliations:** 1 Ludwig Boltzmann Institute Applied Diagnostics, Vienna General Hospital, Währiger Gürtel 18-20, 1090 Vienna, Austria; 2 Faculty of Chemistry, Insititute of Inorganic Chemistry, University of Vienna, Josef-Holaubek-Platz 2, 1090 Vienna, Austria; 3 Rotop Pharmaka GmbH, Bautzner Landstraße 400, 01328 Dresden, Germany; 4 Joint Applied Medicinal Radiochemistry Facility, University of Vienna and Medical University of Vienna, 1090 Vienna, Austria; 5 Division of Nuclear Medicine, Department of Biomedical Imaging and Image- Guided Therapy, Medical University of Vienna, Währinger Gürtel 18-20, 1090 Vienna, Austria

**Keywords:** Dual-tracer SPECT, Indium-111, Technetium-99m, Bone scintigraphy, PSMA scan

## Abstract

**Background:**

Many high-risk prostate cancer (PCa) patients need to undergo ^99m^Tc-based bone scintigraphy as part of conventional diagnostics to assess metastasis to the bone. Besides, targeting the prostate-specific membrane antigen (PSMA) has proven to be very effective for theranostic approaches in PCa in recent years. Therefore, we recognized the high clinical potential of a dual-tracer approach, simultaneously targeting PSMA-expressing lesions and PSMA-negative bone anomalies. In a preclinical PCa model, we aimed at concurrent administration and dual-isotope single-photon emission computed tomography (SPECT) of two radiotracers labeled either with Indium-111 (^111^In) or Technetium-99m (^99m^Tc). Separate depiction of the signals should illustrate the different biodistribution of [^111^In]In-PSMA-I&T and ^99m^Tc-3,3-diphosphono-1,2-propanodicarboxylic acid ([^99m^Tc]Tc-DPD) in the animal.

**Results:**

Temporally parallel radiosyntheses and analysis of [^111^In]In-PSMA-I&T and [^99m^Tc]Tc-DPD were successfully established. Co-incubation studies were conducted with the radioligand stock solutions as well as in vitro experiments using LNCaP cells. LNCaP xenograft-bearing CB17-SCID mice were injected almost simultaneously with [^111^In]In-PSMA-I&T and [^99m^Tc]Tc-DPD and scanned at different time points after injection (2–3 h and 23 h p.i.) on a Siemens Inveon *µ*SPECT/CT. Thereafter, mice were subjected to biodistribution studies. Co-incubation experiments revealed no mutual detrimental effects on radioligand purities. In vitro studies confirmed that the presence of DPD has no influence on [^111^In]In-PSMA-I&T binding and internalization into PSMA-expressing LNCaP cells. By application of three different acquisition windows (^99m^Tc: 126–154 keV, ^111^In: 221–269 keV, open: 80 - 270 keV) radionuclide-specific uptake could be observed and distinguished in the PSMA-expressing xenografts ([^111^In]In-PSMA-I&T) and in the bones ([^99m^Tc]Tc-DPD). Biodistribution data corroborated these results.

**Conclusions:**

Dual-tracer SPECT imaging of PSMA-positive xenografts and PSMA-negative bone structures with [^111^In]In-PSMA-I&T and [^99m^Tc]Tc-DPD, respectively, was successfully realized in this preclinical approach. The positive results of this proof-of-concept study indicate a potentially prompt and successful transfer to clinical settings in which PSMA^−^ bone lesions can be identified besides the PSMA^+^ primary tumor and metastases in a single SPECT/CT scan. This in turn could reduce scan time, CT dose and costs per patient, and provide important insights into tumor biology and improve stratification and treatment of PCa patients.

**Graphical abstract:**

**Figure:**
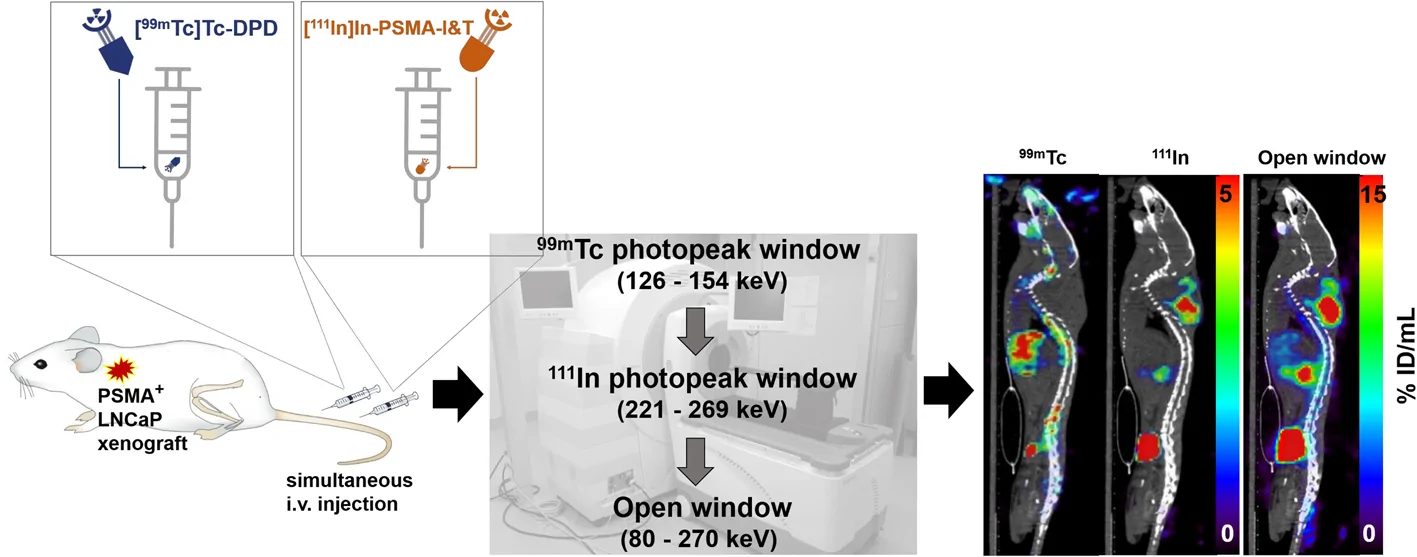

**Supplementary Information:**

The online version contains supplementary material available at https://doi.org/10.1186/s41181-026-00484-6.

## Introduction

Prostate cancer (PCa) can develop to a heterogeneous malignancy, caused either by temporal tumor evolution or by certain therapeutic interventions such as androgen deprivation therapy (ADT) or targeted α-therapy (TAT) (Lawal et al. 2021, Hofman and Emmett 2019, Afshar-Oromieh et al. 2018, Bakht et al. 2018). Thus, expression of the prostate-specific membrane antigen (PSMA) as a PCa-specific biomarker may vary substantially and proves ineffective to be used as a target for radiodiagnostic or -therapeutic interventions in a subset of patients. PSMA is absent or expressed at very low levels in 3–10% of primary tumors with up to 42% PSMA-negative cases for metastatic castration-resistant PCa (mCRPC), as determined *via* immunohistochemistry (Silver et al. 1997, Mannweiler et al. 2009, Minner et al. 2011, Mulati et al. 2025). This resulted in 22–32% of mCRPC patients being excluded from clinical trials of [^177^Lu]Lu-PSMA-617-based radioligand therapy (RLT), due to uniformly low uptake of [^68^Ga]Ga-PSMA-11 or lesions with low [^68^Ga]Ga-PSMA-11 uptake and discordant [^18^F]FDG-avid disease (Emmett et al. 2019, Thang et al. 2019). Low or absent uptake of radiolabeled PSMA ligands in a subset of tumors is likely to limit the prognostic and predictive significance of PSMA-targeted radioligands as standalone agents in advanced PCa (Kouchkovsky et al. 2020, Alberts et al. 2020, Noto et al. 2018). 

Importantly, PCa tends to metastasize to the bone (bone metastases found in 32% of 1589 PCa patients examined *post mortem*), wherefore conventional diagnosis usually includes bone scintigraphy (Mohseninia et al. 2024, Bubendorf et al. 2000). For this purpose, the patient receives a planar 2D whole-body scan after administration of a ^99m^Tc-labeled bone seeking agent (e.g. [^99m^Tc]Tc-MDP). Although cost-effective and widely available, this procedure tends to give false positive results, as certain skeletal anomalies (e.g. degenerative joint diseases, fractures, infections, and benign bone tumors) are characterized by radiotracer uptake as well (Mohseninia et al. 2024, Rager et al. 2017). For a notable reduction of ‘false positives’ and hence higher specificity, the superiority of SPECT/CT over planar imaging has been demonstrated (Rager et al. 2017, Buck et al. 2008, Wieder et al. 2012, Arvola et al. 2022). 

PSMA-based diagnostic procedures outperform these classical planar and SPECT/CT-based bone scans in the majority of PCa patients, as they allow the detection not only of bone metastases, but also recurrent primary tumors as well as proximal and distant extraosseous metastases (e.g. in the lymph nodes) (Hofman et al. 2020, Fendler et al. 2019, Rathke et al. 2018, Zhang et al. 2022, Kabunda et al. 2021, Pyka et al. 2016). However, certain patients (often heavily pretreated by ADT, chemo- or radiation therapy) display a considerable amount of PSMA-negative bone metastases (up to 56% Silver et al. 1997), which can be detected by bone seeking radiotracers instead (Lawal et al. 2021, Mohseninia et al. 2024, Rathke et al. 2018, Uprimny et al. 2018). 

Therefore, we aimed at combining differently radiolabeled diagnostic agents, which specifically bind to PSMA-expressing (PSMA^+^) tumor lesions and PSMA-negative (PSMA^−^) bone lesions of PCa. Provided that the signals emitted by the different imaging probes remain distinguishable, this dual-tracer imaging approach could improve lesion detection and characterization and allow for quantification of intrapatient and intratumoral heterogeneity. Thus, our dual-tracer imaging approach can be used in future studies to collect empirical data to enable improved patient stratification (e.g. early vs. late stage or non-aggressive vs. aggressive disease) and hence, can facilitate treatment decisions. This represents a particular advantage in comparison to other combinatory concepts, like radiolabeled heterobivalent peptidic ligands (HBPL) (Judmann et al. 2020). Dual-tracer imaging offers the possibility to gain complementary information about two distinct biological processes simultaneously, with images perfectly registered both spatially and temporally, significantly reduced scan time for the patient, improved patient comfort and reduced effective dose, as only one CT acquisition is performed (usually yielding 0.1–8.6 mSv, depending on the used device and body region to be scanned) (Bailey and Willowson 2022, Fakhri 2012, Du et al. 2014, Esquinas et al. 2018). 

Application of two different SPECT nuclides that emit γ-rays with clearly distinguishable energies allows for separate depiction after simultaneous administration of the corresponding radiotracers. The image can be either reconstructed by choosing an energy window that includes the photopeaks of both radioisotopes (higher sensitivity, since more lesions are detectable) or the image can be reconstructed by choosing the respective energy windows of a single radioisotope, providing separate information about the distribution of the different radiotracers. In this context, the development of cadmium-zinc-telluride(CZT)-based detectors for SPECT cameras enabled an increased energy resolution when compared to classical thallium-doped sodium iodide (NaI(Tl))-based detectors (Wagenaar ) and thus, further promoted the development of several dual-tracer concepts for SPECT/CT. (Bellevre et al. (2015, Blaire (2018, Caobelli 2016, D’Estanque et al. 2017, Makita 2016)

However, only very few dual tracer-based imaging approaches have yet been initiated in patients for an improved diagnosis of prostate cancer (QUINTANA 2000, Alberts et al. 2023). These approaches (^111^In-labeled capromab pendetide/^99m^Tc-labeled red blood cells (RBCs) and [^68^Ga]Ga‑PSMA‑11/[^18^F]FDG) resulted in higher sensitivity and improved stratification of the patients, but dual-tracer concepts for a separate detection of PSMA^+^ lesions and PSMA^−^ bone anomalies have not yet been reported.

In this study, we focused on dual-tracer SPECT imaging of PSMA^+^ tumor lesions and PSMA^-^ bone structures in a preclinical prostate cancer model with two different SPECT radionuclides that emit γ-rays with distinctly different energies. ^111^In and ^99m^Tc were selected, since their physical properties (^111^In ◊ ^111m^Cd (100% EC) ◊ ^111^Cd (IT, 172 keV (I_γ_: 91%), 245 keV (I_γ_: 94%)), t_1/2_ = 2.80 d; ^99m^Tc ◊ ^99^Tc (99.996% IT; 140.5 keV (I_γ_: 89%)), t_1/2_ = 6.01 h) ( https://www-nds.iaea.org/relnsd/vcharthtml/VChartHTML.html accessed on October 29, 2025., Wadas et al. 2010, Alfassi et al. 2005) allow for separate depiction on a preclinical Siemens Inveon SPECT/CT device after simultaneous administration of the corresponding radiotracers (Giammei et al. 2025). The (pre)clinically well characterized radioligands [^111^In]In-PSMA-I&T and [^99m^Tc]Tc-DPD were selected to target a PSMA^+^ LNCaP xenograft and PSMA^−^ bone structures, respectively, facilitating potential clinical implementation. It should be noted that [^99m^Tc]-DPD-positive uptake may identify bone lesions that are not detected on PSMA imaging but it does not, by itself, indicate PSMA-negative tumor biology, as [^99m^Tc]Tc-DPD accumulation primarily reflects osteoblastic activity and bone remodeling rather than tumor-specific uptake. [^111^In]In-PSMA-I&T is an established tracer for imaging and radio-guided surgery of PSMA^+^ lesions and already entered a Phase II clinical trial (NCT04300673, registered February 17, 2020) (https:/. /clinicaltrials.gov/ accessed on November 01, 2025., Rauscher et al. 2016, Schottelius et al. 2015). [^99m^Tc]Tc-DPD represents an established tracer for bone scintigraphy and has already been approved by the European Medicines Agency (EMA) ( https://www.ema.europa.eu/en/human-regulatory-overview/post-authorisation/data-medicines-iso-idmp-standards-post-authorisation/public-data-article-57-database accessed on March 20, 2026., Fogelman 1987, Buell et al. 1982). Since many high-risk PCa patients are evaluated for metastatic disease either *via* bone scintigraphy or PSMA-targeted SPECT/PET, the transfer of this combinatory dual-tracer SPECT concept to human application seems expedient and readily achievable. Therefore, we aimed at dual-tracer SPECT imaging of PSMA^+^ tumor lesions and PSMA^-^ bone structures in a preclinical PCa model by using [^111^In]In-PSMA-I&T and [^99m^Tc]Tc-DPD, respectively.

## Materials and methods

Detailed information on the synthesis of ^nat^In-PSMA-I&T, [^111^In]In-PSMA-I&T and [^99m^Tc]Tc-DPD, co-incubation studies, in vitro evaluation as well as *in/ex vivo* evaluation is provided in the supporting information (SI). Likewise, details on all methods for synthesis and analysis, on the instruments used, as well as analytical data ((radio-)RP-HPLC, radio-TLC chromatograms as well as mass and γ-spectra, Figures S1-S12) are available in the SI (available at https://link.springer.com/journal/41181).

## Results

### Radiolabeling

Radiochemical purities (RCP) of [^111^In]In-PSMA-I&T at end of synthesis (EOS) were ≥ 95%, as determined *via* radio-RP-HPLC and radio-TLC (*n* = 12, Figure S5 and Figure S7, SI) and the radioligand proved to be stable at room temperature (r.t.) for at least 4 h (RCP ≥ 95% *n* = 4, Figure S9 and Figure S10, SI). The addition of 2-(phosphonomethyl)pentanedioic acid (2-PMPA) for single- or dual-tracer competition studies did not affect RCP (> 95% at EOS, *n* = 5, Table 1). Apparent molar activities (A_m_) at EOS were 52.3 ± 9.9 MBq/nmol (*n* = 17).

For [^99m^Tc]Tc-DPD, RCPs of ≥ 99% (determined *via* radio-TLC, *n* = 9, Figure S11, SI) and specific activities (A_S_) of 0.63 ± 0.1 GBq/mg (*n* = 9) were obtained. According to the manufacturer’s protocol, [^99m^Tc]Tc-DPD can be used up to 8 h after EOS, therefore no additional stability studies were performed ( https://www.thp.at/cms/media/t1918nk.pdf accessed on March 20, 2026). 

[^111^In]In-PSMA-I&T (with or without 2-PMPA) and [^99m^Tc]Tc-DPD were used without any further purification or pH adjustment, since RCPs ≥ 95% were reached for both (radiochemical yield (RCY) > 99%) and pH values at EOS were 5-5.5 and 6, respectively.

## Co-incubation studies

To investigate potential mutual influences on the RCPs, co-incubation experiments were conducted. The pH of the dual-tracer cocktail was determined after mixing equal volumes (5 µL each) of [^111^In]In-PSMA-I&T and [^99m^Tc]Tc-DPD radiopharmaceutical preparation and was found to be 5.5. For further co-incubation studies, one part of the dual-tracer cocktail had to be added in its non-radioactive form, since nuclide-pure analyses were not possible *via* radio-RP-HPLC and radio-TLC. No cross-reactions leading to radioactive by-products or radioactive impurities were detected (Tables 1 and 2).

**Table 1:** Radiochemical purities and pH determined for [^111^In]In-PSMA-I&T after co-incubation over 30 min–1 h with DPD or DPD and 2-PMPA

Co-incubation of [^111^In]In-PSMA-I&T with	30 min incubation				1 h incubation			
	RCP (HPLC) [%]	RCP (TLC) [%]	pH	*n* ^a^	RCP (HPLC) [%]	RCP (TLC) [%]	pH	*n* ^a^
DPD^b^	> 96	> 99	5.5	2	> 97	> 99	5.5	1
DPD^b^ and 2-PMPA	> 97	> 98	5-5.5	2	> 96	> 99	5-5.5	2
	0 min incubation				4 h incubation			
2-PMPA	> 95	> 95	5-5.5	5	≥ 96	> 99	5-5.5	1

^a^n: number of experiments. ^b^Non-radioactive surrogate for [^99m^Tc]Tc-DPD. The exact compositions of the co-incubation solutions are described in the SI.

**Table 2:** Radiochemical purities and pH determined for [^99m^Tc]Tc-DPD after co-incubation over 30 min–1 h with PSMA-I&T or PSMA-I&T and 2-PMPA

Co-incubation of [^99m^Tc]Tc-DPD with	30 min incubation			1 h incubation		
	RCP (TLC) [%]	pH	*n* ^a^	RCP (TLC) [%]	pH	*n* ^a^
PSMA-I&T solution^b^	> 98	5.5	2	> 99	5.5	1
PSMA-I&T solution^b^ and 2-PMPA	> 99	5-5.5	2	> 98	5-5.5	2

^a^n: number of experiments. ^b^Non-radioactive surrogate for [^111^In]In-PSMA-I&T. The exact compositions of the co-incubation solutions are described in the SI.

In vitro evaluation.

**Fig. 1:**
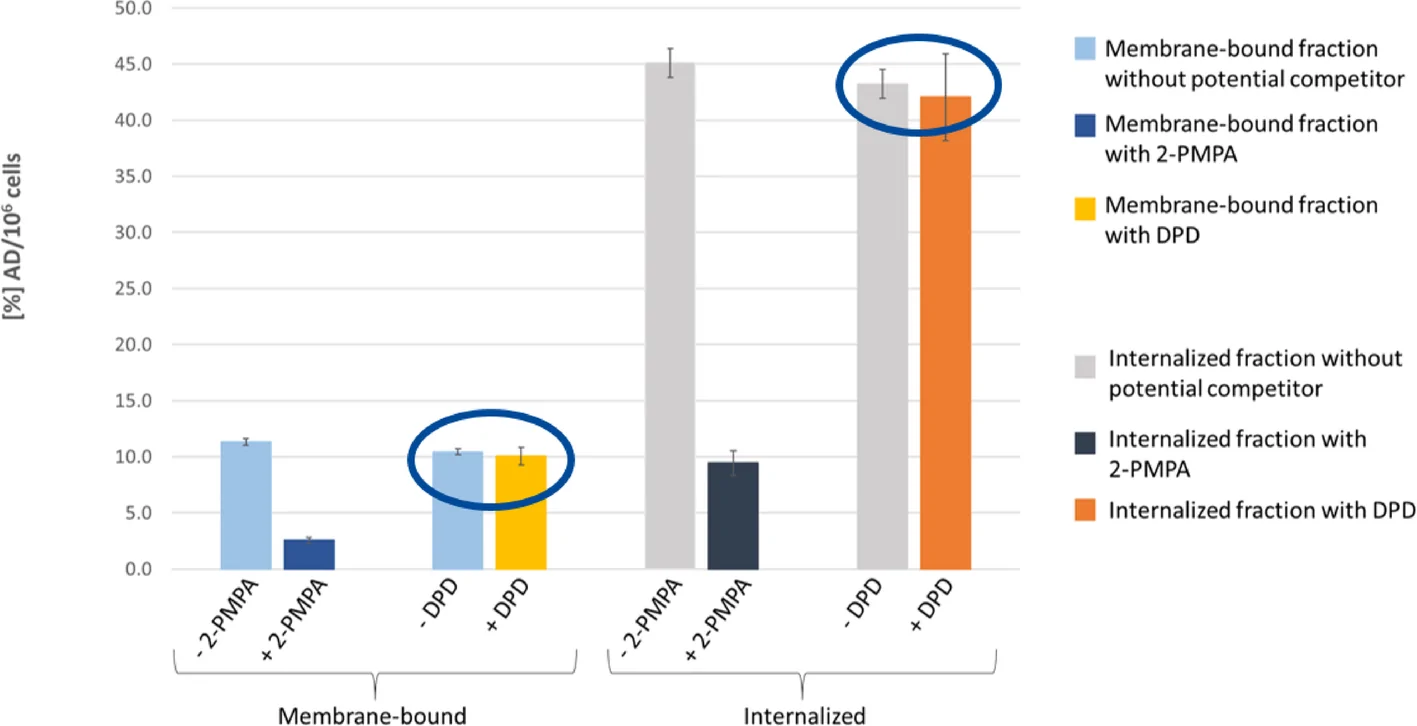
Membrane-binding and internalization of [^111^In]In-PSMA-I&T in PSMA^+^ LNCaP cells with or without (potential) competitor. 2-PMPA (1000-fold excess) acts as high-affinity competitor and considerably decreases radioligand binding to the cell membrane and internalization (blue and dark blue column). By contrast, radioligand binding to the cell membrane and internalization remains unchanged when co-incubated with DPD (1000-fold excess; blue circles around light blue and yellow column as well as around gray and orange column). The amount of radioactivity of each fraction was calculated as percentage of the applied dose (AD) and normalized to 1 × 10^6^ cells (*n* = 3 in triplicate)

To assess mutual effects on radioligand binding, co-incubation studies were performed using LNCaP cells. While addition of the high-affinity PSMA-inhibitor 2-PMPA (Kozikowski et al. 2001)(1000-fold excess) led to considerable reduction of [^111^In]In-PSMA-I&T membrane binding and internalization, no notable change of [^111^In]In-PSMA-I&T binding or uptake could be observed in PSMA^+^ LNCaP cells in the presence of DPD (1000-fold excess) (Fig. 1).

***In/ex vivo*** **evaluation**.

In total, 7 animals were used for *µ*SPECT/CT and biodistribution studies in LNCaP xenograft-bearing CB17-SCID mice. Thereof, 4 were used for dual-tracer studies (3× dual-tracer, 1× dual-tracer + PSMA-competitor) and 3 were used for single-tracer studies (2× single-tracer, 1× single-tracer + PSMA-competitor). Hence, this study represents an initial, exploratory technical proof-of-concept rather than a statistically powered preclinical efficacy study.

**Fig. 2:**
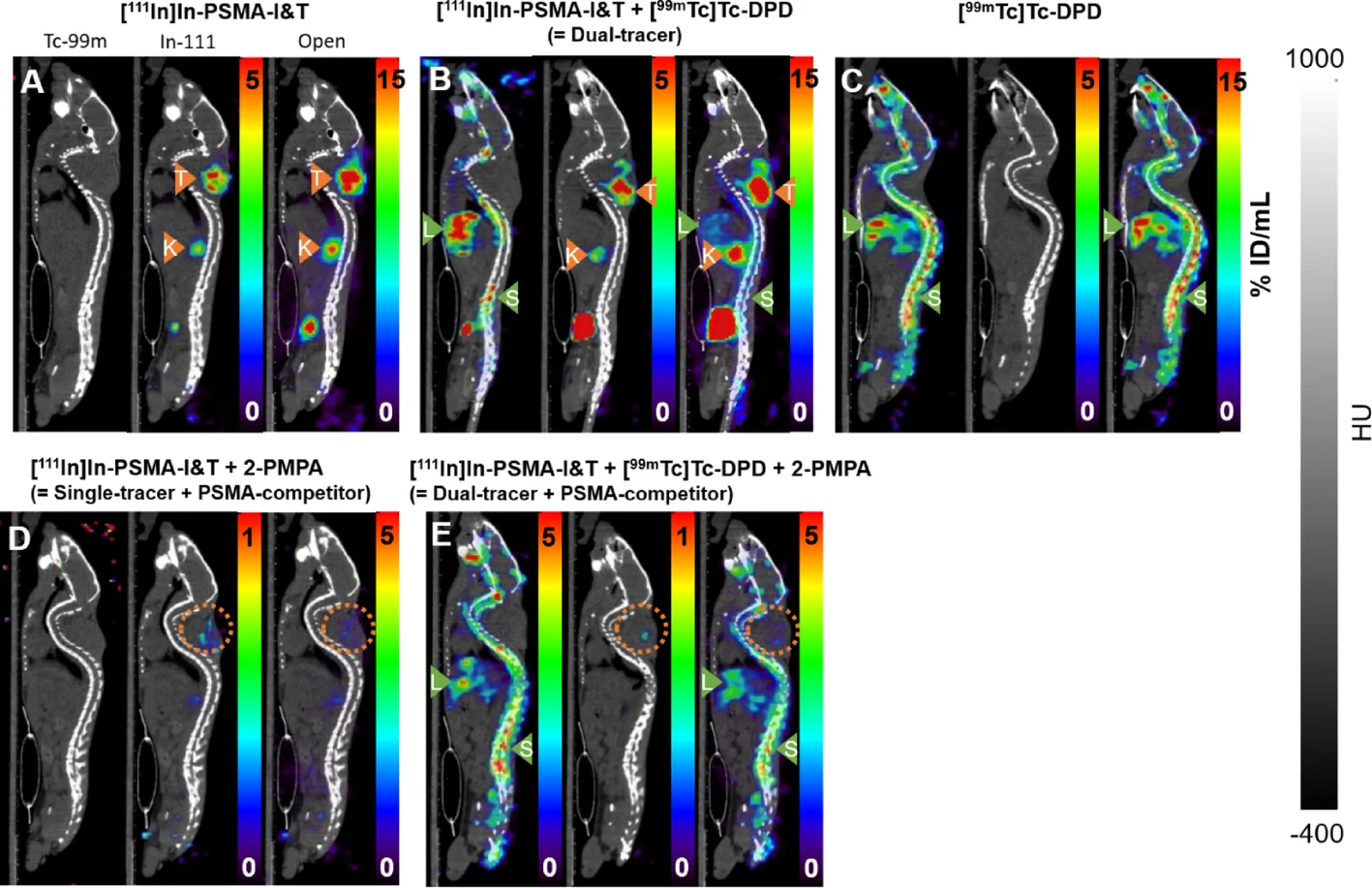
Representative sagittal *µ*SPECT/CT image slices of LNCaP xenograft-bearing CB17-SCID mice, acquired at 2–3 h p.i. and injected with **A** [^111^In]In-PSMA-I&T (31.2 MBq, 1 nmol),** B** [^111^In]In-PSMA-I&T and [^99m^Tc]Tc-DPD (= dual-tracer; 48.4 MBq, 1 nmol and 45.2 MBq, 0.1 mg, respectively),** C** [^99m^Tc]Tc-DPD (54.4 MBq, 0.1 mg),** D** [^111^In]In-PSMA-I&T and 2-PMPA (= single tracer + PSMA-competitor; 44.2 MBq, 1 nmol) and **E** [^111^In]In-PSMA-I&T, [^99m^Tc]Tc-DPD and 2-PMPA (= dual-tracer + PSMA-competitor; 40.9 MBq, 1 nmol and 42.2 MBq, 0.1 mg, respectively). 2-PMPA was co-administered in 1000-fold excess (1 µmol) in (D) and (E). As indicated in (C) and also applicable to all other subsections, uptake values are given in % ID/mL. In addition (A), every left image corresponds to the scan, acquired by using the ^99m^Tc window (126–154 keV), every middle image corresponds to the scan, acquired by using the ^111^In window (221–269 keV) and every right image corresponds to the scan, acquired by using the open window (80–270 keV). For clear comparison, images were scaled to the same maximum uptake value where possible and practical. In (A), (B), (C) and (D), ^99m^Tc and ^111^In window images were scaled to the same maximum uptake value and therefore no additional scale bars were added. Orange arrowheads indicate predominant [^111^In]In-PSMA-I&T uptake in the LNCaP tumor (T) and Kidneys (K), while green arrowheads indicate predominant [^99m^Tc]Tc-DPD uptake in the spine (S) and liver (L). Absent [^111^In]In-PSMA-I&T uptake in the LNCaP tumor xenograft is indicated by dotted orange circles in 2-PMPA-co-injected mice. HU: Hounsfield Units

***µSPECT/CT imaging.*** Representative sagittal *µ*SPECT/CT image slices, acquired at 2–3 h p.i., show the biodistribution of either [^111^In]In-PSMA-I&T or [^99m^Tc]Tc-DPD compared to the dual-tracer (Fig. 2). ^111^In (221 - 269 keV) and open window (80 - 270 keV) scans of [^111^In]In-PSMA-I&T demonstrated high activity accumulation in the LNCaP tumor xenograft (as expected (Schottelius et al. 2015)) and in the kidneys, while almost no activity was observed in the ^99m^Tc window (126 - 154 keV). For [^99m^Tc]Tc-DPD, the main activity accumulation was displayed in the spine and liver in ^99m^Tc and open window scans, whereas no signal appeared in the ^111^In window. The dual-tracer images revealed a notably good delineation of tracer uptake, since the main activity accumulation in the ^99m^Tc window was found in the spine and liver, whereas activity uptake in the LNCaP tumor xenograft was almost exclusively visible in the ^111^In window scan. The open window scan showed the combined uptake of both radioligands (LNCaP tumor xenograft, kidneys, spine, liver, bladder). Co-administration of the PSMA-inhibitor 2-PMPA resulted in notably reduced [^111^In]In-PSMA-I&T uptake in the xenograft, while [^99m^Tc]Tc-DPD accumulation remained visually unaffected. Additional images, acquired at 23 h p.i. are depicted in the SI (Figure S13).

*Quantitative evaluation of µSPECT data*. For quantification of [^111^In]In-PSMA-I&T or [^99m^Tc]Tc-DPD uptake, regions of interest (ROIs) with standard size (2 × 2 × 2 mm^3^) were placed within the respective LNCaP tumor xenograft or the lowest lumbar vertebra of the spine and quantitative values were provided by AMIDE (version 1.0.6). The corresponding CT was used to ensure correct anatomical positioning of the ROIs. In biodistribution studies, the femur was dissected to assess bone uptake. The latter was, however, not sufficiently accessible in *µ*SPECT/CT images for reliable quantification. Quantitative *µ*SPECT values of ^111^In- or ^99m^Tc-specific uptake in the LNCaP tumor xenograft and spine are listed in Table 3 (2–3 h p.i.) and Table S1 (23 h p.i.).

**Table 3:** Quantitative evaluation of *µ*SPECT/CT scans conducted at 2–3 h p.i. ^99m^Tc- or ^111^In-specific uptake values (% ID/mL) in the LNCaP tumor xenograft or spine are listed

Compound (injected activity^a^)	LNCaP tumor xenograft [% ID/mL]		Spine [% ID/mL]	
	^99m^Tc window	^111^In window	^99m^Tc window	^111^In window
[^111^In]In-PSMA-I&T (31.2 MBq, single-tracer)	0.0	4.5	0.0	0.1
[^99m^Tc]Tc-DPD (54.4 MBq, single-tracer)	0.0	0.0	3.6	0.0
[^111^In]In-PSMA-I&T (26.3 MBq) & [^99m^Tc]Tc-DPD (30.5 MBq) (dual-tracer #1)	0.0	3.5	4.1	0.0
[^111^In]In-PSMA-I&T (48.4 MBq) & [^99m^Tc]Tc-DPD (45.2 MBq) (dual-tracer #2)	0.0	6.4	3.9	0.0
[^111^In]In-PSMA-I&T (34.5 MBq) & [^99m^Tc]Tc-DPD (56.1 MBq) (dual-tracer #3)	0.0	5.6	4.0	0.0
[^111^In]In-PSMA-I&T (44.2 MBq) + 2-PMPA (single tracer + PSMA-competitor)	0.0	0.3	0.0	0.0
[^111^In]In-PSMA-I&T (40.9 MBq) & [^99m^Tc]Tc-DPD (42.2 MBq) + 2-PMPA (dual-tracer + PSMA-competitor)	0.0	0.2	3.1	0.0

^a^at time point of injection; Quantitative values for the open window could not be determined since, for technical reasons, the open window was only calibrated to ^111^In (more details given in Sect.  2.5.3 in the SI).

**Fig. 3:**
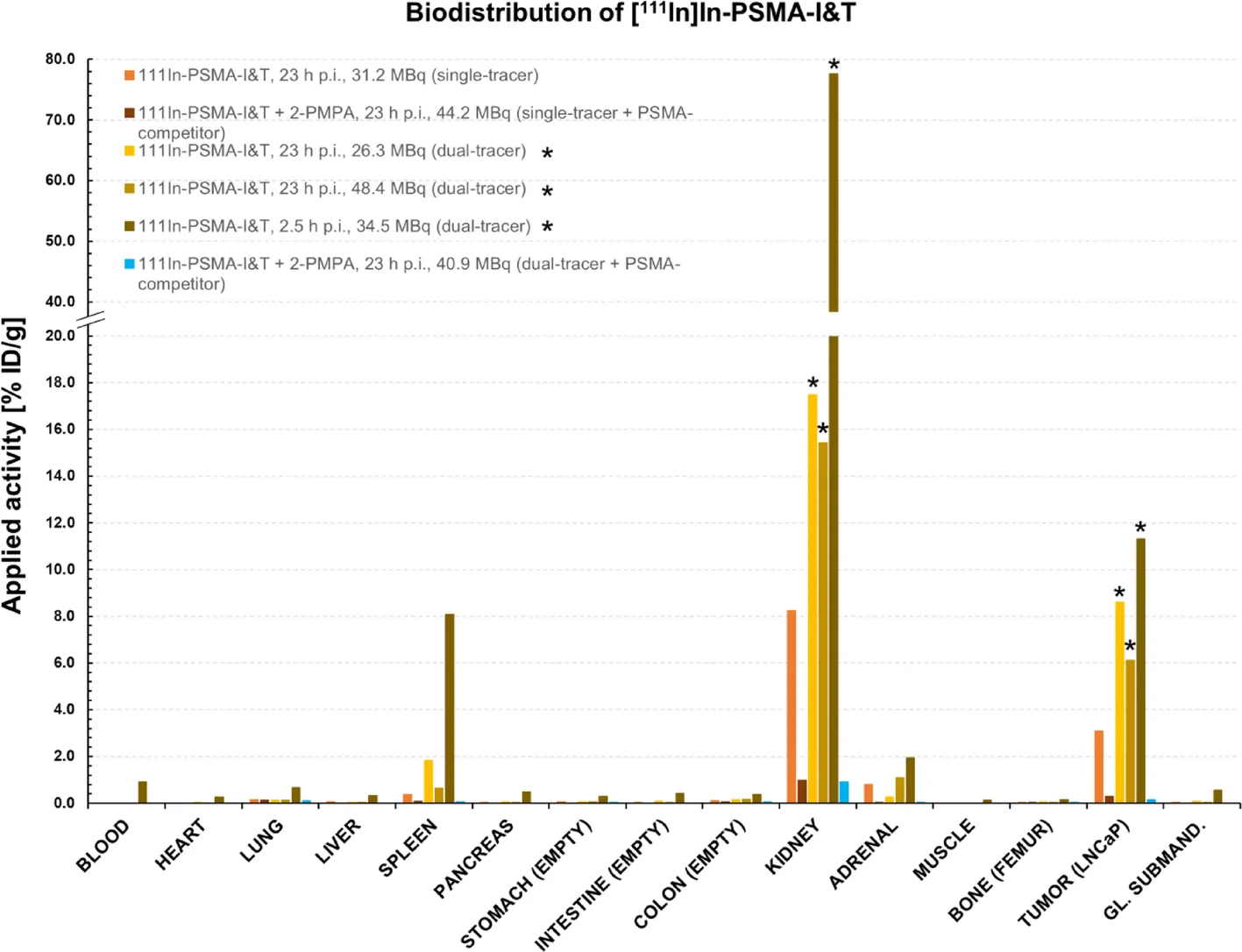
Biodistribution data (% ID/g) of [^111^In]In-PSMA-I&T (26.3–48.4 MBq, 1 nmol) in LNCaP tumor xenograft-bearing CB17-SCID mice. Animals were euthanized at 23 h p.i., with the exception of one animal (2.5 h p.i., dark olive-green bars), that deceased unexpectedly during the second SPECT scan (^111^In window), thus leading to distinctly different tracer uptake in certain organs, such as the spleen (8.09% ID/g), which is mouse-strain specific (Schottelius et al. 2015). Single-tracer data are depicted in orange and dark orange, while dual-tracer data are depicted in yellow, olive, dark-olive and blue. *Blood and organ values of dual-tracer animals were determined as previously described (Giammei et al. 2025), but for tumor (LNCaP) and kidney the procedure was slightly modified. More detailed information on the calculation methods is provided in the supporting information (Sect.  2.6, Figure S14). The exact % ID/g values used for preparing this graph are listed in the SI (Table S2). Gl. submand.: Glandula submandibularis

*Biodistribution studies*. Biodistribution data of [^111^In]In-PSMA-I&T (Fig. 3) corroborated the imaging profile, revealing high ^111^In-activity levels in the LNCaP tumor xenograft (3.08–11.3% ID/g for dual- and single-tracer mice at 2.5 and 23 h p.i.) and kidneys (8.25–77.6% ID/g for dual- and single-tracer mice at 2.5 and 23 h p.i.). Co-injection of the PSMA-competitor 2-PMPA (1000-fold excess) led to a notable reduction of activity uptake in the LNCaP tumor xenograft (0.16 - 0.29% ID/g) and kidneys (0.90 - 0.98% ID/g) of the single- and dual-tracer animal. In all animals, low ^111^In-activity levels were detected in the bone (0.03–0.16% ID/g).

**Fig. 4:**
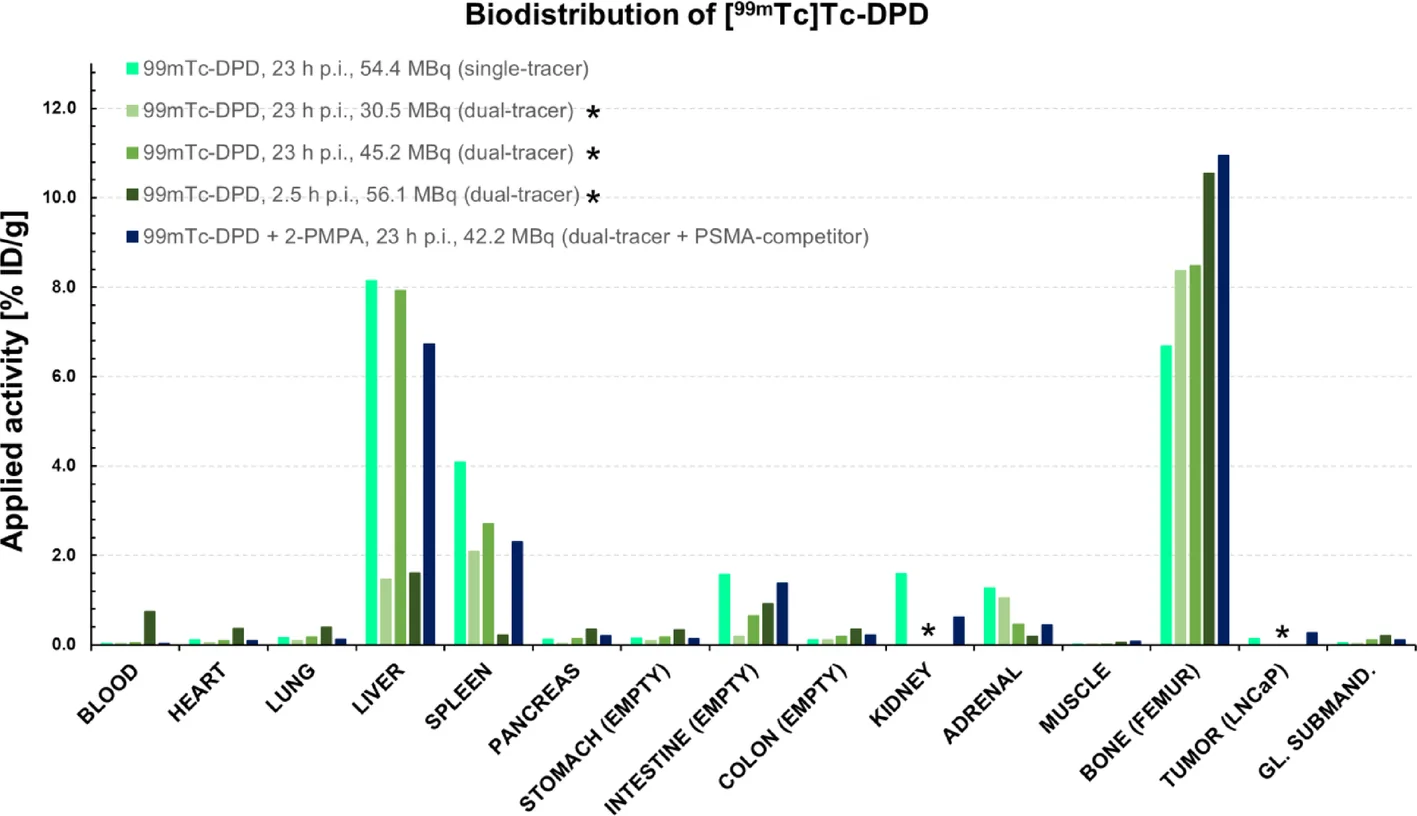
Biodistribution data (% ID/g) of [^99m^Tc]Tc-DPD (30.5 - 56.1 MBq, 0.10 mg) in LNCaP tumor xenograft-bearing CB17-SCID mice. Animals were euthanized at 23 h p.i., with the exception of one animal (2.5 h p.i., dark green bars), that deceased unexpectedly during the second SPECT scan (^111^In window). Data obtained from the single-tracer animal is depicted in turquoise, while dual-tracer data are depicted in gray, green, dark green and dark blue. Blood and organ values of dual-tracer animals were determined as previously described Giammei et al. (2025). *Activity in tumor (LNCaP) and kidney could be not determined (n.d.) due to detector saturation by In-111. More detailed information on the calculation methods is provided in the supporting information (Sect.  2.6, Figure S14). The exact % ID/g values used for preparing this graph are listed in the SI (Table S3). Gl. submand.: Glandula submandibularis

Also for [^99m^Tc]Tc-DPD, biodistribution data (Fig. 4) corroborated the imaging profile, revealing high ^99m^Tc-activity levels in the bone (6.69–11.0% ID/g for dual- and single-tracer mice at 2.5 and 23 h p.i.) and for three animals in the liver (6.72–8.15% ID/g for dual- and single-tracer mice at 23 h p.i.). Co-injection of the PSMA-competitor 2-PMPA (1000-fold excess) had no notable effect on [^99m^Tc]Tc-DPD uptake in the blood or any organ of the dual-tracer animal (*n* = 1). In all animals, low ^99m^Tc-activity levels were detected in the LNCaP tumor xenograft (< 0.27% ID/g) and in the kidneys (< 1.59% ID/g).

## Discussion

[^111^In]In-PSMA-I&T and [^99m^Tc]Tc-DPD were readily accessible *via* standard labeling procedures in high RCP (≥ 95%; RCY > 99%) as well as in high apparent A_m_ (52.3 ± 9.9 MBq/nmol for [^111^In]In-PSMA-I&T) and A_S_ (0.63 ± 0.1 GBq/mg for [^99m^Tc]Tc-DPD). Radiosyntheses were optimized to ensure that a final amount of approximately 40 MBq of the respective radioligand was injected into the animal (~ 80 MBq for dual-tracer animals) in order to obtain sufficient image quality with the Siemens Inveon *µ*SPECT/CT. Stability of both radiotracers at r.t. over a prolonged period (≥ 95% RCP at 4 h after EOS for [^111^In]In-PSMA-I&T determined in this study and usability of [^99m^Tc]Tc-DPD for up to 8 h as specified in the manufacturer’s protocol) is important for dual-tracer logistics, e.g. in case that parallel synthesis of radiotracers is not possible or a delay occurs between radioligand preparation and intravenous (i.v.) injection. The final clear, colorless radiotracer solutions were ready to use for i.v. injection in mice (pH range: 5–8).

Co-incubation studies and in vitro experiments with [^111^In]In-PSMA-I&T and [^99m^Tc]Tc-DPD revealed no mutual detrimental effects on RCPs (Tables 1 and 2*)* or binding and uptake of [^111^In]In-PSMA-I&T in PSMA^+^ LNCaP cells (Fig. 1). According to these positive results and previous phantom measurements with ^111^In and ^99m^Tc performed on the Siemens Inveon *µ*SPECT/CT^(43)^, dual-tracer *µ*SPECT/CT scans at 2–3 h p.i. revealed virtually nuclide-pure depiction of radioligand uptake when reconstructed by using the ^111^In (221–269 keV) or ^99m^Tc photopeak window (126–154 keV). A combined display of radiotracers was achieved by using the open window protocol (80–270 keV, Fig. 2). The *µ*SPECT/CT observations could be corroborated by quantitative *µ*SPECT evaluation (Table 3 and TableS1) and biodistribution studies at 23 h p.i. (Figures 3 and 4). Our *in/ex vivo* experiments, however, should not be interpreted as a preclinical efficacy study with sufficient statistically power; rather they represent an initial, exploratory proof of technical feasibility. As far as it is possible to draw conclusions from this small cohort of animals (1–3 animals per group), it can be stated that the results of single- and dual-tracer mice in *µ*SPECT/CT and biodistribution experiments were consistent. With the exception of 2-PMPA-coinjected mice, the main uptake of [^111^In]In-PSMA-I&T was found in the LNCaP tumor xenograft (PSMA-mediated uptake) and in the kidneys (PSMA-mediated uptake and excretion organ), while the main uptake of [^99m^Tc]Tc-DPD was found in the spine, other bone structures (hydroxyapatite-/Ca^2+^-mediated uptake) and liver (non-specific diffuse liver uptake (Ahmad et al. 2020, Kaye and Hayward 2002)). Competition experiments with 1000-fold excess 2-PMPA confirmed PSMA-specific uptake of [^111^In]In-PSMA-I&T, not only in the single-tracer animal, but also for the dual-tracer experiment. Single-tracer images substantiated a virtually nuclide-pure depiction of radioligands, as no activity was observed in the ^99m^Tc window, when only [^111^In]In-PSMA-I&T (with or without PSMA-competitor) was injected. Likewise, no activity was detected in the ^111^In window, when only [^99m^Tc]Tc-DPD was administered (Fig. 2; Table 3).

It needs to be stated, that our animal studies do not provide a true preclinical model of PSMA^+^ tumor lesions and PSMA^−^ bone metastases, since we did not establish bone metastases in mice. We aimed at keeping the animal model as simple as possible, not only to avoid potential pain exposure and to reduce stress and anxiety for the animals, but also to reduce the potential risk of non-interpretable results and poor study outcome, in case the animal model reveals to be unsuitable (Giammei et al. 2025). Nonetheless, we consider the results of this study as reliable and clearly transferable to bone lesion detection, since [^99m^Tc]Tc-DPD has already been approved by the EMA (https://www ) and it is routinely used in PCa patients for bone scintigraphy.

Apart from clinical applications (^111^In-labeled capromab pendetide/^99m^Tc-labeled RBCs (QUINTANA 2000) and [^68^Ga]Ga‑PSMA‑11/[^18^F]FDG^(39)^), our group recently reported on the combined use of [^111^In]In-RM2 and [^99m^Tc]Tc-PSMA-I&S in dual-xenograft NOD SCID mice, addressing disease-related changes in the expression of PSMA and the gastrin-releasing peptide receptor (GRPR) (Giammei et al. 2025). However, separate detection of PSMA^+^ lesions and PSMA^−^ bone lesions in a dual-tracer SPECT approach has not yet been described. To our knowledge, this study is the first report on a combined use of [^111^In]In-PSMA-I&T and [^99m^Tc]Tc-DPD in a preclinical mouse model (LNCaP xenograft-bearing CB17-SCID) for dual-tracer SPECT imaging of PCa.

The application of [^111^In]In-PSMA-I&T and [^99m^Tc]Tc-DPD in a dual-tracer SPECT approach ideally enables non-invasive and separate quantification of PSMA^+^ lesions and PSMA^−^ bone anomalies. Patients demonstrating intense [^111^In]In-PSMA-I&T uptake would be suitable candidates for PSMA radioligand therapy (e.g. with [^177^Lu]Lu-PSMA-617), whereas those demonstrating reduced [^111^In]In-PSMA-I&T uptake, but enhanced [^99m^Tc]Tc-DPD uptake, may benefit from radioligand palliative bone therapy (e.g. with [^223^Ra]RaCl_2_) (Jadvar 2023). 

In comparison to other dual-tracer concepts, our approach allows for a direct transfer and implementation into clinical routine, as both tracers [^111^In]In-PSMA-I&T and [^99m^Tc]Tc-DPD have been and are still used in nuclear medicine facilities for PCa diagnosis. Hence, the infrastructural workflow only needs to be slightly adapted to provide both tracers in sufficient amount at the same time. We assume that the tracer formulations don’t need to be adjusted, only the administration protocol (approximately simultaneous i.v. injection) and, in particular, the technical requirements need to be established for separate, nuclide-pure image reconstruction and processing or a combined depiction, if necessary. As the optimum imaging time points of both tracers are already known, we assume that no major adjustments or evaluation studies are needed and recommended optimum imaging time points can be adopted.

Despite certain limitations, mainly related to the occasionally limited specificity of bone-seeking agents (e.g. additional detection of benign skeletal anomalies) and to non-detectable extra-osseous metastases (Mohseninia et al. 2024, Rager et al. 2017, Zhang et al. 2022), an overall benefit for the PCa patients by application of such a dual-tracer SPECT approach can be expected, due to the complementary properties of [^111^In]In-PSMA-I&T and [^99m^Tc]Tc-DPD as PSMA-targeted and bone-seeking compounds, respectively. Depending on the outcome of further preclinical and future first in-human studies, it would be possible to determine more precisely which high-risk PCa patients (suspected PSMA-low/PSMA-negative bone lesions, advanced disease or heavily pretreated PCa) are preferably eligible for this dual-tracer SPECT-based diagnostic approach.

## Conclusions

In this proof-of-concept study, we demonstrated the successful application of [^111^In]In-PSMA-I&T and [^99m^Tc]Tc-DPD in a preclinical dual-tracer *µ*SPECT/CT approach. Simultaneous administration of radioligands, but nuclide-specific reconstruction of *µ*SPECT/CT images enabled separate, virtually nuclide-pure depiction of PSMA^+^ tumor lesions and PSMA^−^ bone structures in LNCaP tumor xenograft-bearing CB17-SCID mice. The results were corroborated by quantitative analysis of the *µ*SPECT scans and by biodistribution studies. Aiming at simultaneous detection, but separate depiction of PSMA^+^ tumor and PSMA^−^ bone lesions in PCa patients, this study is an important step towards improved diagnosis of PCa, which exhibits a high propensity to metastasize to the bone. Our concept allows for a direct transfer and implementation into clinical routine, since [^111^In]In-PSMA-I&T and [^99m^Tc]Tc-DPD are routinely used for PCa diagnosis. Their transfer to clinical dual-tracer SPECT/CT imaging could potentially reduce scan time (with concomitant increased capacity of the scanner system), CT dose and costs per patient. In addition, such a dual-tracer SPECT/CT approach will support nuclear medicine physicians in identifying intratumoral and intrapatient heterogeneity, in disease assessment, and realizing a more personalized and effective treatment for the patient.

## Supplementary Information

Below is the link to the electronic supplementary material.

Supplementary Material 1

## Data Availability

The datasets supporting the conclusions of this article are included within this article and its additional file. Further inquiries can be directed to the corresponding authors.

## Abbreviations

2-PMPA: 2-(Phosphonomethyl)pentanedioic acid
AD: Applied dose
ADT: Androgen deprivation therapy
Am: Molar activity
AS: Specific activity
CT: Computed tomography
CZT: Cadmium-zinc-telluride
DPD: 3,3-Diphosphono-1,2-propanedicarboxylic acid, tetrasodium salt
EC: Electron capture
EMA: European Medicines Agency
EOS: End of synthesis
FDG: 2-Fluoro-2-deoxy-D-glucose
Gl. submand.: Glandula submandibularis
GRPR: Gastrin-releasing peptide receptor
HBPL: Heterobivalent peptidic ligands
HU: Hounsfield Units
ID: Injected dose
IT: Isomeric transition
i.v.: Intravenous
mCRPC: Metastatic castration-resistant prostate cancer
MDP: Methylene diphosphonate
PCa: Prostate cancer
PET: Positron emission tomography
p.i.: *Post injectionem*
PSMA: Prostate-specific membrane antigen
RBCs: Red blood cells
RCP: Radiochemical purity
RCY: Radiochemical yield
RLT: Radioligand therapy
RP-HPLC: Reversed-phase high-performance liquid chromatography
SCID: Severe combined immunodeficiency
SI: Supporting Information
SPECT: Single-photon emission computed tomography
TAT: Targeted α-therapy
TLC: Thin-layer chromatography
