# Supplementary material for: Preclinical application of a dual-tracer SPECT approach with [111In]In-PSMA-I&T and [99mTc]Tc-DPD for improved diagnosis of prostate cancer
Source: EJNMMI Radiopharm Chem. 2026 Aug 14;11:82. doi: 10.1186/s41181-026-00484-6 (PMC13768493; doi:10.1186/s41181-026-00484-6)
Supplement: Supplementary file 1 [file 41181_2026_484_MOESM1_ESM.docx]

**Preclinical application of a dual‑tracer SPECT approach with [^111^In]In‑PSMA‑I&T and [^99m^Tc]Tc-DPD for improved diagnosis of prostate cancer**

**- Supporting Information -**

# 1 **MATERIALS**

Solvents and all other general chemicals were purchased from B. Braun, Sigma-Aldrich, Merck, and Carl Roth in the quality grade “for synthesis”. Buffers and stock solutions were prepared using Millipore water unless otherwise stated. Protein LoBind^®^ tubes from Eppendorf were used for syntheses/storage. Research grade PSMA‑I&T was obtained from piCHEM Forschungs- und Entwicklungs GmbH (Raaba‑Grambach, Austria). TECEOS^®^ kit vials (14.23 mg, thereof 13 mg 3,3-diphosphono-1,2-propanedicarboxylic acid, tetrasodium salt (DPD)) were kindly provided by Curium (Petten, Netherlands). [^99m^Tc]NaTcO_4_ was eluted in 5 mL 0.9% NaCl from a MON.TEK ^99^Mo/^99m^Tc generator (30 GBq; Eczacıbaşı-Monrol Nuclear Products Co., Istanbul, Turkey). [^111^In]InCl_3_ was purchased from Curium (Petten, Netherlands). All cell culture reagents were purchased from Thermo Fisher Scientific (Vienna, Austria) or Sigma Aldrich (Vienna, Austria) unless stated otherwise. The blocking reagent 2‑(phosphonomethyl)pentanedioic acid (2‑PMPA) used for *in vitro* studies was obtained from Sigma Aldrich (Vienna, Austria). Cultrex^TM^ Basement Membrane Extract (BME), Type 3, Pathclear (catalog number: 3632-005-02) was obtained from R&D systems Inc. (Minneapolis, USA)

Analytical reversed‑phase high-performance liquid chromatography (RP‑HPLC) was performed by using a Synergi Fusion-RP (C18, 4 µm, 250×4.6 mm) column (Phenomenex, Aschaffenburg, Germany) connected to a VWR Hitachi Chromaster system (Hitachi High-Tech Corporation, Hitachinaka, Japan), comprising a Chromaster 5160 Pump, Chromaster 5310 Column Oven and Chromaster 5410 UV Detector.

Radiolabeled peptides were analyzed by radio‑RP‑HPLC using the same VWR Hitachi Chromaster system, a Synergi Fusion-RP column (C18, 4 µm, 250×4.6 mm) and an Elysia Raytest Gabi Nova radiodetector (Elysia-raytest GmbH, Straubenhardt, Germany) connected to the outlet of the Chromaster 5410 UV Detector.

HPLC solvents were H_2_O (A) and ACN (B) each supplemented with 0.1% trifluoroacetic acid (TFA). Individual gradients, flow, retention times (t*_R_*), and wavelength (λ) are mentioned in the text.

Low resolution (LR)‑MS was performed on a Bruker amaZon speed ETD supplied with ESI ion source and 3D ion trap. High-Resolution Electrospray Ionization (ESI) mass spectra (HR‑MS) were recorded on a Bruker maXis UHR‑TOF spectrometer (Bruker Corporation, Billerica, USA).

Radio‑thin‑layer chromatography (radio-TLC) for [^111^In]In‑PSMA‑I&T was performed on instant thin‑layer chromatography paper impregnated with silica gel (ITLC‑SG, Agilent Technologies Inc., Folsom, USA). Trisodium citrate dihydrate (0.1 M, aq., adjusted to pH 5 with 30% HCl) was used as mobile phase for detection of free ^111^In^3+^ (R*_f_*(^111^In^3+^) = 0.9‑1, R*_f_*([^111^In]In‑PSMA‑I&T) = 0‑0.1). For detection of colloidal ^111^In, NH_4_OAc (1 M, aq.)/MeOH (1/1, *v/v*) was used as mobile phase (R*_f_*(^111^In‑colloid) = 0, R*_f_*([^111^In]In‑PSMA‑I&T) = 0.9‑1). Mobile phases were used for max. 21 days. [^99m^Tc]Tc‑DPD was analyzed on Whatman 1 chromatography paper (Cytiva Europe GmbH, Freiburg im Breisgau, Germany) using methyl ethyl ketone (MEK) as mobile phase for quantification of free ^99m^TcO_4_^−^ (*R*_f_ (^99m^TcO_4_^−^) = 0.9‑1, *R*_f_([^99m^Tc]Tc‑DPD) = 0‑0.1). Whatman 31 ET chromatography paper (Cytiva Europe GmbH, Freiburg im Breisgau, Germany) with 1 M NaCl (aq.) as mobile phase was used for detection of colloidal ^99m^Tc (*R*_f_(^99m^TcO_2_) = 0, *R*_f_([^99m^Tc]Tc‑DPD) = 0.9‑1). Radio‑TLC strips were analyzed by using a GITA* detector and the accompanying software (Elysia-raytest GmbH, Straubenhardt, Germany).

Radionuclide purity was determined by a LB 2045 γ-spectrometer (Berthold Technologies GmbH & Co.KG, Bad Wildbad, Germany).

Activities of the respective probes obtained from internalization assays and biodistribution studies were measured by a 2480 WIZARD^2^ automatic γ‑counter (PerkinElmer, Waltham, USA).

All operations under sterile conditions were accomplished using a MSC-Advantage biological safety cabinet (Thermo Fisher Scientific Inc., Waltham, USA).

# 2 **METHODS**

## 2.1 **Syntheses**

## 2.1.1 **Synthesis of ^nat^In-PSMA‑I&T**

^nat^In-PSMA‑I&T (^nat^In‑**1**) was prepared according to a previously published procedure with minor modifications.^(1)^ In a 0.5 mL Protein LoBind^®^ tube (Eppendorf SE, Hamburg Germany), InCl_3_ (2 µL, 0.05 M in TraceSELECT^TM^-H_2_O, 100 nmol, 5 eq.,) was added to the precursor PSMA-I&T (20 µL, 1 mM in TraceSELECT^TM^-H_2_O, 20 nmol, 1 eq.). The reaction mixture was heated at 95 °C for 25 min. The pH of the reaction solution was 2‑2.5. No purification of the final product was needed, as the chemical purity reached >95% (chemical yield >99%, n = 8, *Figure S1* and *Figure S2*). ^nat^In-**1** stock solutions were used for co‑injections with [^111^In]In‑**1** (confirmation of radioligand identity, *Figure S5*) and proved to be stable over 7 days when stored at ‑20 °C (chemical purity >95%, n = 3, *Figure S3* and *Figure S4).*

RP-HPLC (20% B, 0‑1 min, 20 ‑ 50% B 1‑15 min, 1 mL/min, λ = 220 nm): t*_R_* = 10.7 min; *k* = 2.6. Calculated monoisotopic mass (C_63_H_88_IInN_11_O_23_): 1608.4; found: m/z = 805.7 [M(^nat^In-**1**) + 2H^+^]^2+^, 1610.4 [M(^nat^In‑**1**) + H^+^]^+^.

## 2.1.2 **Radiosyntheses**

Radiosyntheses are described in the main manuscript. ^111^In is a γ‑emitter with principal energies at 172 keV and 245 keV and a half‑life of 2.80 d. ^99m^Tc is a γ‑emitter (140.5 keV) with a half‑life of 6.01 h.^(2)^ All experiments involving ^99m^Tc and ^111^In were performed in laboratories approved for the handling of the respective radionuclides and appropriate safety procedures were followed all the time to prevent contaminations.

[^111^In]In-PSMA-I&T was prepared according to a previously published procedure(1) with minor modifications. 70 µL (40 ‑ 66 MBq) of [^111^In]InCl_3_ (in 0.02 M HCl^(3)^, pH ≈ 1.7^(3)^, <1 μg/mL, 185 MBq in 0.5 mL, 370 MBq/mL at calibration time point; Curium, Petten, Netherlands) were transferred in a 0.5 mL Protein LoBind^®^ tube and 8 µL of freshly prepared NaOAc*3 H_2_O (1 M in TraceSELECT^TM^‑H_2_O) were added to reach a final pH of ~5.5. Subsequently, 2 µL of PSMA‑I&T (0.5 mM in TraceSELECT^TM^-H_2_O, 1 nmol, 1 eq.) were added and the mixture was heated for 25 min at 95 °C. The radiochemical purity (RCP) was determined using radio‑RP‑HPLC and radio‑TLC. Radioligand identity was confirmed by co-injection of ^nat^In-PSMA‑I&T in HPLC analysis (*Figure S5* and *Figure S6*).

For single and dual‑tracer studies with PSMA‑competitor (blocking experiments), 10 µL 2-PMPA (0.1 M in TraceSELECT^TM^‑H_2_O, 1 µmol, 1000 eq., stored at ‑20 °C) were added to the [^111^In]In‑PSMA‑I&T stock solution as well as 4 µL of freshly prepared 1 M NaOAc*3 H_2_O to reach a pH of 5-5.5. The RCP was determined using radio‑RP‑HPLC and radio‑TLC.

[^99m^Tc]Tc-DPD was prepared and the final solution analyzed according to the manufacturer’s protocol with minor modifications.^(4)^ To avoid frequent exposure to high radioactivity and to have more kits available for ^99m^Tc‑labelings (only preclinical amounts were needed), the TECEOS^®^ (DPD) vials (14.23 mg, thereof 13 mg DPD, Curium) were opened and the content subdivided into 1.78 mg aliquots (thereof 1.625 mg DPD), hereinafter referred to as “kit”. The kits were stored at ‑20 °C under N_2_ atmosphere and consumed no later than 5 weeks after aliquot preparation. For radiosynthesis, 1.1 GBq of the [^99m^Tc]TcO_4_^-^ containing eluate were added to the kit and filled up with 0.9% NaCl to 1 mL. The 1.5 mL Protein LoBind^®^ tube was shaken until everything was dissolved and the solution was incubated for 5 min at r.t. The RCP was determined using radio‑TLC and the radionuclide purity was determined *via* γ‑spectroscopy. The final clear, colorless solution had a pH of ~6.

## 2.2 **HPLC chromatograms, mass spectra and TLC chromatograms**

**^nat^In‑PSMA‑I&T (^nat^In‑1)**

**Figure S1**. HPLC chromatogram of ^nat^In‑PSMA‑I&T (^nat^In‑**1**). t_R_ = 10.7 min (20% B, 0‑1 min, 20‑50% B 1‑15 min, 1 mL/min, λ = 220 nm). Chemical purity: 97.4%.

**Figure S2**. LR-MS data of ^nat^In‑PSMA‑I&T (^nat^In‑**1**).

**Figure S3**. HPLC chromatogram of ^nat^In‑PSMA‑I&T (^nat^In‑**1**) at 7 d after end of synthesis (EOS) and storage at ‑20 °C during that period. t_R_ = 10.8 min (20% B, 0‑1 min, 20‑50% B 1‑15 min, 1 mL/min, λ = 220 nm). Chemical purity: 97.8%.

**Figure S4**. LR-MS data of ^nat^In‑PSMA‑I&T (^nat^In‑**1**) at 7 d after EOS and storage at ‑20 °C during that period.

**[^111^In]In‑PSMA‑I&T ([^111^In]In‑1)**

**Figure S5**. Confirmation of identity and purity of [^111^In]In‑PSMA‑I&T ([^111^In]In‑**1**). HPLC chromatogram of ^nat^In‑PSMA‑I&T (^nat^In‑**1**) (front red trace). t_R_ = 10.8 min (20% B, 0‑1 min, 20‑50% B 1‑15 min, 1 mL/min, λ = 220 nm). Chemical purity: >99%. Radio‑HPLC chromatogram of [^111^In]In‑PSMA‑I&T (rear green trace), t_R_ = 10.9 min (20% B, 0‑1 min, 20‑50% B 1‑15 min, 1 mL/min). Radiochemical purity: 97.1%. The short time delay between the peaks results from downstream positioning of the radiodetector.

**Figure S6**. LR-MS data of ^nat^In‑PSMA‑I&T (^nat^In‑**1**) as co-injected with [^111^In]In‑PSMA‑I&T in Figure S5. The ^nat^In‑**1** batch was prepared and analyzed via LR-MS one day prior to co‑injection and stored at ‑20°C until then.

**Figure S7**. Radio‑TLC chromatograms of [^111^In]In‑PSMA‑I&T, analyzed on A) ITLC‑SG chromatography paper using trisodium citrate dihydrate (0.1 M, aq., adjusted to pH 5 with 30% HCl) as mobile phase (RCP >99%) and B) ITLC‑SG chromatography paper with NH_4_OAc (1 M, aq.)/MeOH (1/1, v/v) as mobile phase (RCP >99%).

***Figure S8***. Radio‑HPLC chromatogram of [^111^In]In‑PSMA‑I&T after dilution in 0.9% NaCl (30 µL reaction solution/30 µL 0.9% NaCl) and 30 min incubation. These conditions simulate potential dilution of the radioligand as performed for i.v. injection in mice. t*_R_* = 10.8 min (20% B, 0‑1 min, 20‑50% B 1‑15 min, 1 mL/min). Radiochemical purity: 97.7%.

***Figure S9****.* Radio‑HPLC chromatogram of [^111^In]In‑PSMA‑I&T at 4 h after EOS (storage at r.t.), t_R_ = 10.9 min (20% B, 0‑1 min, 20‑50% B 1‑15 min, 1 mL/min). Radiochemical purity: 95.1%.

**Figure S10**. Radio‑TLC chromatograms of [^111^In]In‑PSMA‑I&T at 4 h after EOS (storage at r.t.), analyzed on A) ITLC‑SG chromatography paper using trisodium citrate dihydrate (0.1 M, aq., adjusted to pH 5 with 30% HCl) as mobile phase (RCP >99%) and B) ITLC‑SG chromatography paper with NH_4_OAc (1 M, aq.)/MeOH (1/1, v/v) as mobile phase (RCP >99%).

**[^99m^Tc]Tc‑DPD ([^99m^Tc]Tc-2)**

**Figure S11**. Radio‑TLC chromatograms of [^99m^Tc]Tc‑DPD, analyzed on A) Whatman 1 chromatography paper using MEK as mobile phase (RCP >99%) and B) Whatman 31 ET chromatography paper with 1 M NaCl (aq.) as mobile phase (RCP >99%).

***Figure S12****.* γ-spectrum of [^99m^Tc]Tc‑DPD.

## 2.3 **Co-incubation studies**

For co-incubation experiments with [^111^In]In‑PSMA‑I&T, a non-radioactive surrogate for [^99m^Tc]Tc‑DPD was prepared. Instead of adding 1 mL of ^99m^Tc-generator eluate (eluent: 0.9% NaCl), 1 mL 0.9% NaCl was added to the kit. An aliquot (30 ‑ 60µL) was taken and mixed with the same volume of [^111^In]In‑PSMA‑I&T stock solution (20 - 50 MBq, with or without 2‑PMPA). After incubation for 30 min or 1 h, the mixtures were analyzed *via* radio‑RP‑HPLC and radio‑TLC and their pH was measured.

For co-incubation experiments with [^99m^Tc]Tc-DPD, a non-radioactive surrogate for [^111^In]In‑PSMA‑I&T was prepared. 70 µL of ^nat^InCl_3_ (4.52 µM = 1 µg/mL, pH ≈ 1.7(3)) were transferred in a 0.5 mL Protein LoBind^®^ tube and 8 µL of freshly prepared NaOAc*3 H_2_O (1 M in TraceSELECT^TM^‑H_2_O) were added to reach a pH of ~5.5. Subsequently, 2 µL of PSMA‑I&T (0.5 mM in TraceSELECT^TM^-H_2_O, 1 nmol, 1 eq.) were added. In case of additional co-incubation with the PSMA‑competitor, 2‑PMPA was added to this PSMA‑I&T solution as described above. An aliquot (30 ‑ 60µL) was taken and mixed with the same volume of [^99m^Tc]Tc-DPD stock solution (20 - 40 MBq). After incubation for 30 min or 1 h, the mixtures were analyzed *via* radio‑TLC and their pH was measured.

As [^111^In]In-PSMA-I&T and [^99m^Tc]Tc-DPD were used in dual-tracer SPECT imaging experiments, co‑incubation studies were conducted to simulate commingling of the radioligands when injecting i.v. into mice *via* one catheter. Although, all radioligands could be injected *via* separate catheters (substantially facilitates calculation of A_0,In-111_ and A_0,Tc-99m_ of blood and dissected organs) and in quick succession (within 5 min), we conducted co-incubation studies, in case mixing of tracers would become necessary, due to obstruction of one of the catheters and hence only one open i.v. access available.

## 2.4 ***In vitro* evaluation**

### 2.4.1 **Cell culture**

LNCaP cells were purchased from ATCC (CRL‑1740^TM^, Lot: 70042941) and cultivated in DMEM/F‑12 (1/1)+GlutaMAX^TM^ medium (Gibco REF: 31331‑093) supplemented with 10% heat-inactivated fetal bovine serum (FBS), 100 units/mL penicillin and 100 μg/mL streptomycin. Cells were kept in a humidified atmosphere (37 °C, 5% CO_2_). Accutase^®^ solution was applied to detach the cells for cell passaging or harvesting. One day (24 ± 2 h) prior to *in vitro* experiments, LNCaP cells were harvested and centrifuged at 1200 rpm for 4 min at room temperature (ROTANTA 460 RC floorstanding centrifuge, Andreas Hettich GmbH, Tuttlingen, Germany). After centrifugation, the supernatant was disposed and the cell pellet was resuspended in culture medium. An aliquot (10 µL) was taken, mixed with Trypan Blue solution (0.4%, Thermo Fisher Scientific Inc.,Waltham,MA, USA) in a 1/1 (*v/v*) ratio and cells were counted with a LUNA^TM^ automated cell counter (Logos Biosystems, Villeneuve d’Ascq, France). For internalization experiments 1 × 10^6^ cells/well were seeded in poly‑L‑lysine (PLL)-coated 6‑well plates (Greiner Bio‑One GmbH, Kremsmünster, Austria). Cells were regularly checked for mycoplasma contamination, and only passages proven to be free from mycoplasma were used for *in vitro* and *in vivo* experiments. All *in vitro* experiments were carried out with radiolabeled peptides providing a radiochemical purity of ≥95% (radio‑RP‑HPLC).

### 2.4.2 **Internalization studies**

The culture medium was removed and 1.30 mL of Ham’s F‑12 Nutrient Mix supplemented with 1% FBS was added to each well (1 × 10^6^ LNCaP cells/well). After incubation for 1 h at 37 °C (5% CO_2_), 100 µL of [^111^In]In‑PSMA-I&T (2 nM in 0.9% NaCl, final concentration: 0.13 nM/well, ~10 kBq/well) was added (n = 6) as well as 100 µL phosphate-buffered saline (PBS) for determination of total binding (n = 3) or 100 µL of (potential) blocking reagent (2 µM in 0.9% NaCl, n = 3). 2‑PMPA or DPD were used as (potential) blocking reagents in separate experiments (final concentration: 133 nM/well) and the final assay volume was 1.5 mL/well. Cells were incubated for 2 h at 37 °C (5% CO_2_) and subsequently processed as previously described^(5)^ to obtain free, membrane‑bound and internalized fractions of the radioligand. The amount of radioactivity of each fraction was quantified in a γ‑counter, calculated as percentage of the applied dose and normalized to 1×10^6^ cells (n = 3 in triplicate).

In order to assess whether [^99m^Tc]Tc‑DPD as co-injected radiotracer has an influence on [^111^In]In‑PSMA-I&T binding and uptake in PSMA^+^ LNCaP cells, we conducted *in vitro* competition experiments with DPD (potential competitor, 1000‑fold excess) and 2‑PMPA (usual blocking agent, known high-affinity PSMA inhibitor^(6)^, 1000‑fold excess). For practical reasons (cumbersome nuclide‑pure analysis of dual‑tracer γ-counter data, as explained in section 2.6.1) and due to the fact, that the main component of administered [^99m^Tc]Tc‑DPD is still unlabeled precursor, we used the content of DPD kits for co‑incubation experiments with [^111^In]In‑PSMA‑I&T.

A vice-versa-experiment, in which the influence of PSMA‑I&T on binding and uptake of [^99m^Tc]Tc‑DPD into the bones would be investigated, was not conducted. We concluded from published *in vivo* experiments, that no notable change in [^99m^Tc]Tc-DPD bone uptake is to be expected in the presence of [^111^In]In‑PSMA-I&T. Wirtz (née Weineisen) *et al*. reported that uptake of [^68^Ga]Ga-/[^177^Lu]Lu‑PSMA-I&T in bones was shown to be considerably low in LNCaP‑tumor bearing CD-1 *nu/nu* mice (0.27 ± 0.08% ID/g at 1 h p.i. for [^68^Ga]Ga‑PSMA‑I&T and 0.22 ± 0.05% ID/g at 1 h p.i. and 0.15 ± 0.02% ID/g at 24 h p.i. for [^177^Lu]Lu‑PSMA-I&T, n = 4) and humans (8.45 × 10^‑3^ ‑ 4.83 × 10^‑3^ mGy/MBq for osteogenic cells, extrapolated from mouse biodistribution data after application of [^177^Lu]Lu-PSMA I&T).^(1, 7)^

## 2.5 ***In/ex vivo* evaluation**

Six-to-eight-week-old male CB17‑SCID mice (CB-17/lcr-Prkdc^scid^/lcrlcoCrl) were obtained from Charles River Laboratories (Sulzfeld, Germany). Animals were kept in individually ventilated cages with 12 h light‑dark cycle. All experiments were approved by the Federal Ministry of Education, Science and Research, Republic of Austria (2021‑0.878.017 and 2023-0.163.191) and performed according to the guidelines from the Federation of the European Laboratory Animal Science Associations (FELASA). After an acclimatization period of two weeks, animals were injected subcutaneously with 8 × 10^6^ LNCaP cells (in 200 μL serum-free culture medium/Cultrex^TM^ BME, Type 3 (1/1, *v/v*)) into the dorsal right shoulder. Tumor growth and body weight were monitored every 48 hours. Animals were subjected to imaging after 4 - 6 weeks of tumor growth (final tumor volume of approximately 250 mm^3^). Endpoints were defined as rapid weight loss up to 20% body weight, tumor volume ≥1000 mm^3^, tumor volume <1000 mm^3^ with signs of necrosis (ulcerated surface), as well as clearly detectable apathy and pain upon touch.

### 2.5.1 ***µ*SPECT/CT imaging**

Xenograft-bearing CB17‑SCID mice were anesthetized using isoflurane (1.5‑2.5%, with an oxygen flow of 1 L/min) and intravenously injected with 26.3 - 48.4 MBq (1 nmol) of [^111^In]In‑PSMA‑I&T and 30.5 ‑ 56.1 MBq (0.1 mg) of [^99m^Tc]Tc-DPD for dual-tracer imaging (n = 3). For single‑tracer imaging, animals were injected with 31.2 MBq (1 nmol) of [^111^In]In‑PSMA‑I&T or 54.4 MBq (0.1 mg) of [^99m^Tc]Tc-DPD (n = 1 each). For competition studies (n = 1 each), 1 µmol of 2‑PMPA (1000‑fold excess with respect to PSMA‑I&T) was coinjected with [^111^In]In‑PSMA‑I&T (44.2 MBq, 1 nmol, single tracer +  PSMA‑competitor) or with 40.9 MBq (1 nmol) of [^111^In]In‑PSMA‑I&T and 42.2 MBq (0.1 mg) of [^99m^Tc]Tc‑DPD (dual‑tracer + PSMA‑competitor). Animals were injected *via* the tail vein (max. injection volume 200 μL) and returned to their cages until the imaging procedure (randomized, non-blinded study design). For dual‑tracer applications, the radioligands were injected *via* separate catheters and in quick succession (within 5 min). SPECT imaging was performed at 2-3 h and 23 h *post injectionem* (p.i.). Animals were placed into the field of view of the scanner (*μ*SPECT/CT Inveon, Siemens Medical Solution, Knoxville, TN, USA) and vital parameters (respiration, body temperature) were continuously monitored using a dedicated device (BioVet; m2m imaging, Cleveland, OH, USA). A 7-minute cone‑beam CT was conducted before each SPECT measurement series. For all animals and imaging time points, the scan protocol included three consecutive static SPECT acquisitions (first: ^99m^Tc photopeak window, second: ^111^In photopeak window, third: open window; 20 min for each SPECT at 2‑3 h p.i., 40 min for each SPECT at 23 h p.i.) using a multi-pinhole collimator (5 × 1 mm). The following energy window settings were set before image acquisition: ^99m^Tc photopeak window (141 keV): 126‑154 keV (quantitative calibration on ^99m^Tc), ^111^In photopeak window (245 keV): 221‑269 keV (quantitative calibration on ^111^In) and open window: 80‑270 keV (quantitative calibration on ^111^In). After the 2‑3 h p.i. imaging time point, mice returned to their cages to be re‑imaged at 23 h p.i. Static *µ*SPECT/CT images at 23 h p.i. were acquired *post mortem* (cervical dislocation under anesthesia) and after cardiac puncture. Further biodistribution studies were performed after the scans. SPECT images were reconstructed using OSEM (6 subsets, 10 views per detector, 16 iterations) and corrected for attenuation (based on the μ‑map generated from the CT), scatter, and decay. Image processing and quantitative evaluation was performed using the image analysis software AMIDE (version 1.0.6).

**Figure S13:** Representative sagittal μSPECT/CT image slices of LNCaP xenograft-bearing CB17‑SCID mice, acquired at 23 h p.i. and injected with (A) [^111^In]In‑PSMA‑I&T (31.2 MBq, 1 nmol), (B) [^111^In]In‑PSMA‑I&T and [^99m^Tc]Tc‑DPD (= dual‑tracer; 48.4 MBq, 1 nmol and 45.2 MBq, 0.1 mg, respectively), (C) [^99m^Tc]Tc‑DPD (54.4 MBq, 0.1 mg), (D) [^111^In]In‑PSMA‑I&T and 2‑PMPA (= single tracer + PSMA‑competitor; 44.2 MBq, 1 nmol) and (E) [^111^In]In‑PSMA‑I&T, [^99m^Tc]Tc‑DPD and 2‑PMPA (= dual‑tracer + PSMA‑competitor; 40.9 MBq, 1 nmol and 42.2 MBq, 0.1 mg, respectively). 2‑PMPA was co‑administered in 1000‑fold excess (1 µmol) in (D) and (E). As indicated in (C) and also applicable to all other subsections, uptake values are given in % ID/mL. In addition (A), every left image corresponds to the scan, acquired by using the ^99m^Tc window (126‑154 keV), every middle image corresponds to the scan, acquired by using the ^111^In window (221‑269 keV) and every right image corresponds to the scan, acquired by using the open window (80‑270 keV). For clear comparison, images were scaled to the same maximum uptake value where possible and practical. In (A), (C) and (D), ^99m^Tc and ^111^In window images were scaled to the same maximum uptake value and in (B) all acquisition windows were scaled to the same maximum uptake value, wherefore no additional scale bars were added. Orange arrowheads indicate predominant [^111^In]In‑PSMA‑I&T uptake in the LNCaP tumor (T) and Kidneys (K), while green arrowheads indicate predominant [^99m^Tc]Tc‑DPD uptake in the spine (S) and liver (L). Absent [^111^In]In‑PSMA‑I&T uptake in the LNCaP tumor xenograft is indicated by dotted orange circles in 2-PMPA‑co-injected mice. HU: Hounsfield Units.

### 2.5.2 **Quantitative evaluation of *µ*SPECT data**

For quantification of [^111^In]In‑PSMA‑I&T or [^99m^Tc]Tc‑DPD uptake, regions of interest (ROIs) with standard size (2×2×2 mm^3^) were placed within the respective LNCaP tumor xenograft or the lowest lumbar vertebra of the spine and quantitative values were provided by AMIDE (version 1.0.6). The corresponding CT was used to ensure correct anatomical positioning of the ROIs. In biodistribution studies, the femur was dissected to assess bone uptake. The latter was, however, not sufficiently accessible in *µ*SPECT/CT images for reliable quantification.

**Table S1:** Quantitative evaluation of µSPECT/CT scans conducted at 23 h p.i. ^99m^Tc‑ or ^111^In‑specific uptake values (% ID/mL) in the LNCaP tumor xenograft or spine are listed.

| Compound (injected activity^a^) | LNCaP tumor xenograft [% ID/mL] | | Spine [% ID/mL] | |
| --- | --- | --- | --- | --- |
|  | ^99m^Tc window | ^111^In window | ^99m^Tc window | ^111^In window |
| [^111^In]In‑PSMA‑I&T (31.2 MBq, single‑tracer) | 0.0 | 3.1 | 0.0 | 0.0 |
| [^99m^Tc]Tc-DPD (54.4 MBq, single‑tracer) | 0.0 | 0.0 | 2.3 | 0.0 |
| [^111^In]In‑PSMA‑I&T (26.3 MBq) & [^99m^Tc]Tc‑DPD (30.5 MBq)  (dual-tracer #1) | 0.0 | 2.9 | 0.0 | 0.0 |
| [^111^In]In‑PSMA‑I&T (48.4 MBq) & [^99m^Tc]Tc‑DPD (45.2 MBq)  (dual‑tracer #2) | 0.0 | 4.2 | 0.0 | 0.0 |
| [^111^In]In‑PSMA‑I&T (34.5 MBq) & [^99m^Tc]Tc‑DPD (56.1 MBq)  (dual‑tracer #3) | n.d.^b^ | n.d.^b^ | n.d.^b^ | n.d.^b^ |
| [^111^In]In-PSMA-I&T (44.2 MBq) + 2‑PMPA (single tracer + PSMA‑competitor) | 0.0 | 0.2 | 0.0 | 0.0 |
| [^111^In]In‑PSMA‑I&T (40.9 MBq) & [^99m^Tc]Tc‑DPD (42.2 MBq) + 2‑PMPA  (dual‑tracer + PSMA‑competitor) | 0.0 | 0.0 | 3.1 | 0.0 |

^a^at time point of injection; ^b^this animal deceased unexpectedly already at 2.5 h p.i.,(during the second SPECT scan, i.e. ^111^In window), thus quantitative *µ*SPECT values at 23 h p.i. could be not determined (n.d.) for this mouse. Quantitative values for the open window could not be determined since, for technical reasons, the open window was only calibrated to ^111^In (more details given in section 2.5.3).

### 2.5.3 **Technical limitations of dual-tracer *µ*SPECT/CT on the Siemens Inveon device**

Early imaging time points for dual‑tracer animals are denoted as 2-3 h p.i. since three 20‑min‑SPECT scans were conducted consecutively (^99m^Tc photopeak window at 2 h p.i. 🡪 ^111^In photopeak window at 2.5 h p.i. 🡪 open window at 3 h p.i.) after the CT. These sequential scans were performed, as SPECT data could not be acquired in list‑mode format. For technical reasons, specific photopeak windows needed to be chosen ahead and the respective calibration factor (CF), either for ^111^In or ^99m^Tc was directly applied to the acquired dataset. As a result, data from one scan could not be processed into different images by choosing different energy windows after image acquisition. Although these technical constraints did not allow for a true simultaneous SPECT of [^111^In]In-PSMA‑I&T and [^99m^Tc]Tc-DPD, *µ*SPECT/CT images provided a visual display of *in vivo* biodistribution of radiotracers within a narrow time frame of 1.5 p.i. and nuclide‑pure depiction in dual‑tracer animals was possible (*Figure 2*, main manuscript). At 23 h p.i., animals were sacrificed to stop *in vivo* biodistribution and to generate a single imaging time point, despite several consecutive scans.

The aforementioned technical limitations also led to the situation, that the open window scans only gave quantitative correct results for animals, in which only [^111^In]In-PSMA-I&T (with or without 2‑PMPA) was administered. Also for the open window protocol, a specific CF was directly applied to the acquired data set and in this case, ^111^In was used to set this CF. Hence, minimum and especially maximum color bar thresholds for the open window as stated in *Figure 2* (main manuscript) and *Figure S13* are only valid to a very limited extent, as quantitatively correct results could not be obtained for [^99m^Tc]Tc‑DPD single‑tracer and dual‑tracer images. Nevertheless, the open window images could still be used for qualitative evaluation and revealed a combined display of [^111^In]In-PSMA-I&T and [^99m^Tc]Tc‑DPD uptake, indicating a higher sensitivity for lesion detection when both tracers are injected. These technical constraints apply specifically to the preclinical imaging device used (Siemens Inveon), but can be avoided for (pre)clinical scanners in case list‑mode acquisition is possible.

## 2.6 **Biodistribution studies**

Animals used in imaging studies were sacrificed prior to the last imaging time point (23 h p.i.) by cervical dislocation under isofluorane anesthesia. Blood was immediately collected by cardiac puncture. After the final *µ*SPECT/CT scan, selected organs were removed and weighed, and organ activities were measured in a γ-counter. For animals in which a dual‑tracer cocktail was administered, the organs were measured several times over a period of 6‑8 weeks to use these datasets in specific calculation methods established previously^(8)^ to obtain the correct activity for each individual radionuclide at the start of the measurement series (t = 0). More detailed information on the calculation of A_0,In-111_ and A_0,Tc‑99m_ of dual‑tracer probes is given in the following section 2.6.1. Deviations between imaging (% ID/mL) and biodistribution data (% ID/g) could be observed due to the lower sensitivity of the *µ*SPECT/CT imaging device (Siemens Inveon) when compared to the γ-counter (2480 WIZARD^2^ automatic γ-counter) used.

### 2.6.1 **Calculation of A_0,In‑111_ and A_0,Tc‑99m_ of dual‑tracer samples**

The procedure for the measurement of dual‑tracer biodistribution probes and the evaluation of the respective γ‑counter data has already been described previously, with the following equations used(8):

$A_{t}=A_{0}\cdot e^{-\frac{\ln\left( 2 \right)}{t_{\frac{1}{2}}} \cdot t}$	(1)

$\frac{A_{t}}{A_{0}}= e^{\frac{- ln(2)}{t_{\frac{1}{2}}} \cdot t}$	I $ln$	(2)

$ln(\frac{A_{t}}{A_{0}})= \frac{- ln(2)}{t_{\frac{1}{2}}} \cdot t$	(3)

$\ln\left( A_{t} \right)-ln(A_{0})= \frac{- ln(2)}{t_{\frac{1}{2}}} \cdot t$	(4)

$\ln\left( A_{t} \right)=-\frac{\ln\left( 2 \right)}{t_{\frac{1}{2}}}\cdot t+\ln(A_{0})$	(5)

**slope	y-intercept**

The resulting linear equation (data selection criteria for the graph: Δt > 10 × t_1/2_(^99m^Tc) ≈ 60 h, at least 5 data points) provides a slope, which should be as close as possible to the theoretical slope, matching the half-life of ^111^In (*equation 6*). The slope accuracy can be calculated by dividing the slope obtained by the linear equation by the theoretical slope and should be 100 ± 5%.

$-\frac{\ln\left( 2 \right)}{t_{\frac{1}{2}}}=-\frac{\ln\left( 2 \right)}{2.805 \cdot24 h}= -0.0103$	(6)

Besides, the y-intercept will give the value of ln(A_0_), since at t = 0, ln(A_t_) = ln(A_0_) (*equation 5*). By applying the natural exponential function (inverse function of the natural logarithm) on the y-intercept, A_0,In-111_ of the respective organ can be obtained (*equations 7* and *8*).

$e^{\ln(A_{0})}=A_{0}$	(7)

$A_{0, \mathrm{In}111}=e^{y\mathrm{intercept}}$	(8)

Subtraction of A_0,In‑111_ from the total measured counts per minute (CPM) at t = 0 (= A_0,total,meas._) gives A_0,Tc-99m_ (*equation 9*).

$A_{0,Tc-99m}={A_{0, total,meas.}- A}_{0, In-111}$	(9)

As a control, the ln(A_t,Tc-99m_) values can be calculated based on very early time points (^99m^Tc decay dominant), e.g. from Δt = 0 - 10 h and plotted as before (*equation 5*). The resulting slope should give ‑ln(2)/t_1/2_(^99m^Tc) (= ‑0.1154) with an accuracy of 100 ± 5%.

The CPM values thus obtained for ^111^In and ^99m^Tc for each organ/body fluid were corrected to the time point at which the very first probe of the measurement series was measured (t = 0). The CPM values were converted into % of injected dose (ID) based on the applied activity and normalized to the weight of the probe to obtain % ID/g, analogous to single tracer measurements.

In this study, due to high [^111^In]In-PSMA-I&T uptake, the LNCaP tumor xenografts and kidneys showed activity levels that exceeded the linear measurement range of the γ‑counter (dead-time effects) (*Figure S14*) . Consequently, the ^99m^Tc activity could not be calculated because no reliably measured A_0,total,meas._ value was obtained. The [^111^In]In-PSMA-I&T uptake in tumor and kidney could be calculated as extrapolation of the ^111^In reading from the linear range of the detector as described in *equation 8*.

**Figure S14**: CPM data of the kidney of mouse #4 (= dual‑tracer mouse #2) as recorded by the γ‑counter, decay corrected for the decay of ^111^In to time point t=0. Theoretically, the CPMs should follow a horizontal line except for time points earlier than ~10 half‑lifes of ^99m^Tc. In these early frames, the contribution of ^99m^Tc to the CPM readings should be visible as an exponential decay with decay constant y(^99m^Tc)-y(^111^In). The measured data show that for time points <~200 h, detector saturation was reached and dead time effects lead to underestimation of activity. For the late time points >800 h, the contribution of background counts become significant.

### 2.6.2 **Biodistribution data of [^111^In]In-PSMA‑I&T and [^99m^Tc]Tc-DPD**

The values used for generating *Figure 3* and *4* in the main manuscript are listed in *Table S2* and *Table S3*.

| Organ | Mouse #1  Single‑tracer (23 h p.i.) | Mouse #2  Single‑tracer + PSMA‑compe-titor (23 h p.i.) | Mouse #3  Dual‑tracer (23 h p.i.) | Mouse #4  Dual‑tracer (23 h p.i.) | Mouse #5  Dual‑tracer (2.5 h p.i.) | Mouse #6  Dual‑tracer + PSMA‑compe-titor (23 h p.i.) |
| --- | --- | --- | --- | --- | --- | --- |
| Blood | 0.01 | 0.00 | 0.02 | 0.01 | 0.92 | 0.00 |
| Heart | 0.02 | 0.01 | 0.03 | 0.02 | 0.25 | 0.01 |
| Lung | 0.14 | 0.13 | 0.14 | 0.13 | 0.67 | 0.10 |
| Liver | 0.07 | 0.02 | 0.04 | 0.04 | 0.33 | 0.02 |
| Spleen | 0.37 | 0.07 | 1.83 | 0.65 | 8.09 | 0.07 |
| Pancreas | 0.03 | 0.01 | 0.05 | 0.04 | 0.49 | 0.01 |
| Stomach (empty) | 0.07 | 0.02 | 0.05 | 0.05 | 0.29 | 0.03 |
| Intestine (empty) | 0.04 | 0.02 | 0.08 | 0.04 | 0.42 | 0.02 |
| Colon (empty) | 0.10 | 0.06 | 0.15 | 0.16 | 0.38 | 0.05 |
| Kidney | 8.25 | 0.98 | 17.5 | 15.4 | 77.6 | 0.90 |
| Adrenal | 0.79 | 0.05 | 0.26 | 1.09 | 1.94 | 0.04 |
| Muscle | 0.01 | 0.00 | 0.01 | 0.01 | 0.12 | 0.00 |
| Bone (femur) | 0.03 | 0.03 | 0.06 | 0.03 | 0.16 | 0.03 |
| Tumor (LNCaP) | 3.08 | 0.29 | 8.60 | 6.12 | 11.3 | 0.16 |
| Glandula  submandibularis | 0.05 | 0.01 | 0.07 | 0.05 | 0.56 | 0.01 |

**Table S2**: Biodistribution data of [^111^In]In-PSMA‑I&T (26.3 - 48.4 MBq, 1 nmol) at 2.5 h (n=1) or 23 h p.i. (n=6), injected in male LNCaP tumor xenograft‑bearing CB17‑SCID mice. Data are expressed as percentage of the injected dose per gram (% ID/g). Single‑tracer: Only [^111^In]In-PSMA-I&T was injected; Dual‑tracer: [^111^In]In-PSMA‑I&T and [^99m^Tc]Tc‑DPD were injected and values for [^111^In]In‑PSMA‑I&T were determined as described in section 2.6.1. Biodistribution time points in brackets refer to [^111^In]In‑PSMA‑I&T.

| Organ | Mouse #7  Single‑tracer (23 h p.i.) | Mouse #3  Dual‑tracer (23 h p.i.) | Mouse #4  Dual‑tracer (23 h p.i.) | Mouse #5  Dual‑tracer (2.5 h p.i.) | Mouse #6  Dual‑tracer + PSMA‑compe-titor (23 h p.i.) |
| --- | --- | --- | --- | --- | --- |
| Blood | 0.03 | 0.03 | 0.05 | 0.74 | 0.03 |
| Heart | 0.11 | 0.04 | 0.10 | 0.36 | 0.09 |
| Lung | 0.17 | 0.09 | 0.18 | 0.39 | 0.12 |
| Liver | 8.15 | 1.47 | 7.93 | 1.61 | 6.72 |
| Spleen | 4.09 | 2.10 | 2.71 | 0.22 | 2.31 |
| Pancreas | 0.13 | 0.03 | 0.14 | 0.35 | 0.20 |
| Stomach (empty) | 0.16 | 0.09 | 0.18 | 0.34 | 0.14 |
| Intestine (empty) | 1.58 | 0.19 | 0.65 | 0.93 | 1.38 |
| Colon (empty) | 0.12 | 0.11 | 0.19 | 0.36 | 0.22 |
| Kidney | 1.59 | n.d.* | n.d.* | n.d.* | 0.63 |
| Adrenal | 1.27 | 1.05 | 0.46 | 0.19 | 0.44 |
| Muscle | 0.01 | 0.01 | 0.01 | 0.06 | 0.09 |
| Bone (femur) | 6.69 | 8.37 | 8.49 | 10.6 | 11.0 |
| Tumor (LNCaP) | 0.13 | n.d.* | n.d.* | n.d.* | 0.27 |
| Glandula submandibularis | 0.05 | 0.03 | 0.11 | 0.21 | 0.11 |

**Table S3**: Biodistribution data of [^99m^Tc]Tc-DPD (30.5 ‑ 56.1 MBq, 0.1 mg) at 2.5 h (n=1) or 23 h p.i. (n=6), injected in male LNCaP tumor xenograft‑bearing CB17‑SCID mice. Data are expressed as percentage of the injected dose per gram (% ID/g). Single‑tracer: Only [^99m^Tc]Tc‑DPD was injected; Dual‑tracer: [^99m^Tc]Tc‑DPD and [^111^In]In‑PSMA‑I&T were injected and values for [^99m^Tc]Tc‑DPD were determined as described in section 2.6.1. Biodistribution time points in brackets refer to [^99m^Tc]Tc‑DPD.

*A_0,Tc‑99m_ in these organs could be not determined by *equation 9*, as no reliably measured A_0,total,meas._ value was obtained due to exceeding the linear measurement range of the γ‑counter.

# 3 **ABBREVIATIONS**

2-PMPA	2-(Phosphonomethyl)pentanedioic acid

ACN	Acetonitrile

aq.	Aqueous

BME	Basement Membrane Extract

CF	Calibration factor

CPM	Counts per minute

DMEM	Dulbecco's Modified Eagle Medium

DPD	3,3-diphosphono-1,2-propanedicarboxylic acid, tetrasodium salt

eq.	Equivalent

FBS	Fetal bovine serum

FELASA	Federation of the European Laboratory Animal Science Associations

HR-MS	High-resolution mass spectrometry

HU	Hounsfield units

ID	Injected dose

LR‑MS	Low-resolution mass spectrometry

OSEM	Ordered subset expectation maximization

PBS	Phosphate-buffered saline

p.i.	*Post injectionem*

PLL	Poly‑L‑lysine

PSMA	Prostate-specific membrane antigen

ROI	Region of interest

RP‑HPLC	Reversed‑phase high-performance liquid chromatography

r.t.	Room temperature

SCID	Severe combined immunodeficiency

TFA	Trifluoroacetic acid

UHR-TOF	Ultra-High-Resolution-Time-of-Flight

# 4 **REFERENCES**

1.	Wirtz M. Development of biomarkers for molecular imaging and endoradiotherapy of prostate cancer [PhD]. Munich: Technical Universtity of Munich; 2015.

2.	<https://www-nds.iaea.org/relnsd/vcharthtml/VChartHTML.html> accessed on October 29, 2025

3.	Giammei C, Jouini N, Brandt MR, Frey S, Mindt TL, Cardinale J. Unexpected transformation of n.c.a. [111In]InCl3 in stock solutions into an unreactive [111In]In-species. Applied Radiation and Isotopes. 2022;180:110037.

4.	<https://www.thp.at/cms/media/t1918nk.pdf> accessed on March 20, 2026

5.	Giammei C, Balber T, Benčurová K, Cardinale J, Berroterán-Infante N, Brandt M, et al. Sorbitol as a Polar Pharmacological Modifier to Enhance the Hydrophilicity of 99mTc-Tricarbonyl-Based Radiopharmaceuticals. Molecules. 2020;25(11):2680.

6.	Kozikowski AP, Nan F, Conti P, Zhang J, Ramadan E, Bzdega T, et al. Design of Remarkably Simple, Yet Potent Urea-Based Inhibitors of Glutamate Carboxypeptidase II (NAALADase). J Med Chem. 2001;44(3):298-301.

7.	Weineisen M, Schottelius M, Wester H-J, Simecek J, Baum RP, Kulkarni HR, et al. 68Ga- and 177Lu-Labeled PSMA I&T: Optimization of a PSMA-Targeted Theranostic Concept and First Proof-of-Concept Human Studies. J Nucl Med. 2015;56(8):1169-76.

8.	Giammei C, Balber T, Felber V, Dillinger T, Cardinale J, Brandt MR, et al. Towards Dual-Tracer SPECT for Prostate Cancer Imaging Using [99mTc]Tc-PSMA-I&S and [111In]In-RM2. Pharmaceuticals. 2025;18(7):1002.
